# Complete Genome Sequence of an Italian Swine Enteric Coronavirus Strain 77590/2019

**DOI:** 10.1128/mra.00386-22

**Published:** 2022-08-16

**Authors:** Alice Papetti, Paolo Bonilauri, Chiara Chiapponi, Laura Baioni, Maria Beatrice Boniotti

**Affiliations:** a Istituto Zooprofilattico Sperimentale della Lombardia ed Emilia-Romagna, Brescia, Italy; b Istituto Zooprofilattico Sperimentale della Lombardia ed Emilia-Romagna, Reggio Emilia, Italy; c Istituto Zooprofilattico Sperimentale della Lombardia ed Emilia-Romagna, Parma, Italy; DOE Joint Genome Institute

## Abstract

In this study, we report the detection of a case of Swine enteric Coronavirus (SeCoV) in Northern Italy. The complete genome sequence of 28,081 nucleotides was obtained. This strain had a genome nucleotide identity of 98.15 to 98.45% with the SeCoV circulating in Europe during 1993-2015, but it also displayed unique genetic features.

## ANNOUNCEMENT

SeCoV is a single-stranded, non-segmented, positive-sense RNA virus of the *Coronaviridae* family, *Alphacoronavirus* genus, that has an approximately 28 kb genome and viral envelopes. This strain is a recombinant Coronavirus (CoV) that circulated in Italy between 2009 and 2012 (1). Most of the SeCoV genome was derived from transmissible gastroenteritis virus (TGEV), but the *S* gene originated from porcine epidemic diarrhea virus (PEDV) (1). This chimeric CoV was also identified in Germany (2012) (2), in Slovakia (2015) (3, 4), and Spain (1993 to 1994, 1998 to 1999 and 2014) (5). SeCoV causes enteritis, as do PEDV and TGEV, but it appears to result in a low piglet mortality rate (1, 4).

In March 2019, a SeCoV strain infecting pigs was detected on a farm located in Modena Province, Northern Italy. The strain produced the clinical sign of diarrhea and resulted in a low piglet mortality rate. The intestinal contents of three suckling pigs were pooled, diluted 1:10 (wt/vol) in minimum essential medium, and submitted for viral RNA extraction as described by Bertasio et al. (6). The presence of the virus was detected by a PEDV real-time reverse transcription-polymerase chain reaction (RT-PCR) assay (6) and the sequencing of the S1 portion of the spike gene (7).

The whole-genome sequence was obtained by amplifying long, overlapping PCR products that covered the complete genome. The protocol and primers used were previously reported in a study by Boniotti and colleagues (1). The sequencing libraries were prepared using the NEXTERA-XT DNA Library Preparation Kit (Illumina Inc., San Diego, CA, USA) and sequenced on an Illumina MiSeq System using the MiSeq Reagent Kit v2, 250-cycle paired-end run (Illumina). The raw reads were trimmed by a CLC Genomic Workbench version 11.0.1 (Qiagen, Milan, Italy) for quality (limit 0.05) and for ambiguous nucleotides (maximum of 2 nucleotides allowed). Moreover, the removal of primer sequences was performed using the previously described list of primers (1). The reads obtained were 189.124, and they were *de novo* assembled using the Geneious algorithm of Geneious Prime version 2020.0.5 (Biomatters Ltd., Auckland, New Zealand) with default parameters. Both the evaluation of nucleotide and amino acid sequence similarity using the identity matrix, and the prediction and annotation of coding DNA sequences (CDS) were performed by Geneious Prime version 2020.0.5, using the default settings. The complete 28,081 nt genome revealed high nucleotide sequence similarity levels with SeCoV strain Italy/213306/2009 (98.45%, accession number (AN) KR061459.1), isolate SeCoV_GER_L00930_2012 (98.36%, AN LT545990.1), and isolate SeCoV-1480-Murcia-Lorca (98.15%, AN MN692770.1). The average genome completeness obtained was 99.9%, with a mean coverage of 862.8 and an average guanine-cytosine (GC) content of 38.14%.

This recombinant coronavirus was called SeCoV strain Italy/77590/2019.

The nucleotide and amino acid (aa) identity levels of the CDSs of the strain Italy/77590/2019 compared with the reference strains of SeCoV presented in GenBank revealed that the strain Italy/77590/2019 has more variation in its *E*, *S*, and *ORF3* genes than do other CDSs (Table 1).

**TABLE 1:** Nucleotide and amino acid identity range (%) of the coding DNA sequences of the SeCoV strain Italy/77590/2019 compared to the SeCoV reference strains available in GenBank

	Nucleotide identity (%)		Amino acid identity (%)	
ORF1a	98.26 to 98.53^*a*^		97.98 to 98.13^*a*^	
ORF1b	98.63 to 98.84^*a*^		99.18 to 99.33^*a*^	
Spike	*1993 to 1999 strains:* 92.66 to 94.17^*b*^	*2009 to 2016 strains:* 96.39 to 97.54^*a*^^,^^*c*^	*1993 to 1999 strains:* 92.42 to 93.21^*b*^	*2009 to 2016 strains:* 95.67 to 96.68^*a*^^,^^*c*^
ORF3b	98.64 to 99.05^*a*^^,^^*c*^		97.13 to 97.95^*a*^^,^^*c*^	
Envelope	95.98 to 96.79^*a*^^,^^*c*^		93.9 to 95.12^*a*^^,^^*c*^	
Membrane	98.23 to 98.73^*a*^^,^^*c*^		98.09 to 98.85^*a*^^,^^*c*^	
Nucleocapsid	97.74 to 98.52^*a*^^,^^*c*^		98.43 to 98.95^*a*^^,^^*c*^	
ORF7	98.31 to 99.58^*a*^^,^^*c*^		98.72^*a*^^,^^*c*^	

a This includes SeCoV Italy/213306/2009 (AN KR061459.1), SeCoV_GER_L00930_2012 (AN LT545990.1), and SeCoV-1480-Murcia-Lorca (AN MN692770.1).

b This includes SeCoV-BU-Burgos (AN MN692757.1), SeCoV-EGV-Segovia (AN MN692758.1), SeCoV-SG1-Segovia (AN MN692759.1), SeCoV-VA-Valladolid (AN MN692760.1), SeCoV-MU2-Murcia (AN MN692761.1), and SeCoV-AYL-Segovia-Ayllon (AN MN692762.1).

c This includes SeCoV 42845 (AN KX689261).

Analysis was performed using the identity matrix in Geneious Prime version 2020.0.5 with the default parameters.

The *ORF3a* gene of the SeCoV Italy/77590/2019 strain contained a 12 nt deletion that included a stop codon, and this leads to a predicted longer *ORF3a*-coding sequence than is found in the other strains. In particular, the *ORF3a* sequence was 135 nt, encoding a protein of 44 aa, while the other SeCoVs possessed 93 nt *ORF3a* sequences that encoded 30 aa proteins, with the exception of isolate SeCoV_GER_L00930_2012, which had an 84 nt sequence that encoded 27 aa proteins.

The genetic characterization of this SeCoV strain revealed its continuous evolution from 1993 to 2019, even though it is not endemic and only sporadic cases have been reported in Europe to date. The geographic origin and spread of the virus are difficult to determine because the discrimination of SeCoV from PEDV/TGEV is commonly not achieved by routine diagnostic tests. This case shows the propensity of this virus to mutate and generate new variants that may pose risks to both animal and human safety.

### Data availability.

The complete genome sequences and raw sequencing reads obtained in this study were deposited under GenBank accession number MT821905 and BioProject accession number PRJNA856142. This BioProject includes three libraries that were deposited in NCBI SRA under accession numbers SRX16042256, SRX16042257, and SRX16042258. Each library contains a different set of amplicons, which together represent the whole genome.
